# Estimation of Isolation Times of the Island Species in the *Drosophila simulans* Complex from Multilocus DNA Sequence Data

**DOI:** 10.1371/journal.pone.0002442

**Published:** 2008-06-18

**Authors:** Shannon R. McDermott, Richard M. Kliman

**Affiliations:** Department of Biological Sciences, Cedar Crest College, Allentown, Pennsylvania, United States of America; University of California, Berkeley, United States of America

## Abstract

**Background:**

The *Drosophila simulans* species complex continues to serve as an important model system for the study of new species formation. The complex is comprised of the cosmopolitan species, *D. simulans*, and two island endemics, *D. mauritiana* and *D. sechellia*. A substantial amount of effort has gone into reconstructing the natural history of the complex, in part to infer the context in which functional divergence among the species has arisen. In this regard, a key parameter to be estimated is the initial isolation time (*t*) of each island species. Loci in regions of low recombination have lower divergence within the complex than do other loci, yet divergence from *D. melanogaster* is similar for both classes. This might reflect gene flow of the low-recombination loci subsequent to initial isolation, but it might also reflect differential effects of changing population size on the two recombination classes of loci when the low-recombination loci are subject to genetic hitchhiking or pseudohitchhiking

**Methodology/Principal Findings:**

New DNA sequence variation data for 17 loci corroborate the prior observation from 13 loci that DNA sequence divergence is reduced in genes of low recombination. Two models are presented to estimate *t* and other relevant parameters (substitution rate correction factors in lineages leading to the island species and, in the case of the 4-parameter model, the ratio of ancestral to extant effective population size) from the multilocus DNA sequence data.

**Conclusions/Significance:**

In general, it appears that both island species were isolated at about the same time, here estimated at ∼250,000 years ago. It also appears that the difference in divergence patterns of genes in regions of low and higher recombination can be reconciled by allowing a modestly larger effective population size for the ancestral population than for extant *D. simulans*.

## Introduction

The study of speciation genetics can be imperfectly divided into two categories: the study of genes that directly contribute to speciation and population genetic analyses that aim to reconstruct the natural historical context in which speciation occurs. In an ever-increasing assemblage of model systems for the study of the genetics of speciation, the *Drosophila simulans* species complex has maintained its prominence, due in part to focused genome sequencing efforts that complement decades of study on the complex and its sister species, *D. melanogaster*.

The *D. simulans* complex is comprised of two island species, *D. mauritiana* and *D. sechellia*, along with cosmopolitan *D. simulans*. Calibrating a molecular clock by the divergence of the complex from *D. melanogaster*, and assuming that this split occurred ∼3 mya [1], prior studies of the *D. simulans* complex indicated that *D. sechellia* and *D. mauritiana* were initially isolated from the mainland species approximately 500 and 250 kya, respectively [2]. The aim of the current study is to better estimate the times at which the incipient island species were first isolated from the ancestor of *D. simulans*. There are two rationales for this work. First, functional divergence among these species, at least in terms of genetic incompatibilities in hybrids, is considerable [3]–[9]. Thus, reliable estimates of isolation times are required to estimate the rate at which functional divergence can evolve in incipient species.

A second rationale relates to prior failure to reject a strict isolation model (SIM) (*i.e.*, no gene flow subsequent to initial isolation) for the members of the *D. simulans* complex [2], [10] that may reflect lack of statistical power. Levels of DNA sequence divergence in genomic regions of high and low recombination are very different within the complex, although their divergences from *D. melanogaster* are similar [10]. It is possible that genes in low recombination regions, especially those on the fourth chromosome, are less diverged within the complex due to introgression subsequent to initial isolation. This would be consistent with the finding that hybrid sterility does not map to this chromosome [11], making introgression more likely if the opportunity were present.

There is, however, an alternative explanation for reduced divergence that can be reconciled with the SIM: a higher effective population size (*N*_e_) in the common ancestor than in extant *D. simulans* coupled with a shallower coalescent for low-recombination genes in the ancestral population. The latter is predicted under various models under which selection on one site also reduces *N*_e_ over an extended region of linked sites. This is perhaps most easily envisioned under Maynard Smith and Haigh's [12] genetic hitchhiking model, whereby the rapid fixation of a new, beneficial mutation reduces local nucleotide diversity at adjacent sites that can not segregate independently due to insufficient opportunity for recombination during the selective sweep. However, other models (*e.g.*, background selection [13], pseudohitchhiking/“genetic draft” [14]) also predict reduced polymorphism in regions of low recombination. For either the hitchhiking or pseudohitchhiking model to dramatically reduce polymorphism in regions of reduced recombination, the frequency of selective sweeps must be high enough to make recent sweeps likely in genomic regions of low recombination. Estimates of the frequency of selective sweeps indicate that these are quite common in Drosophila [15]–[17]. For example, using the estimate of Smith and Eyre-Walker [15] of one adaptive amino acid substitution every ∼45 years in *D. simulans* and *D. yakuba* (assuming 10 generations/year), a selective sweep on the fourth chromosome, which accounts for ∼1.06% of the codons in *D. melanogaster*, would be expected every ∼4250 years.

Here, we present two simple models to estimate isolation times from multilocus data. The first, a 2-parameter model, was used to estimate isolation times separately for loci in regions of high and low recombination, along with a substitution rate correction factor for the island species. This model provides a straightforward approach to estimating isolation times from multilocus data that should be broadly applicable to model and non-model species pairs that include an appropriate outgroup (here, *D. melanogaster*). Given no compelling reason to do otherwise, the analyses were performed under the assumption that the *N*_e_ of the ancestor of the island species and *D. simulans* was the same as that of extant *D. simulans*. Although separate analyses on the two recombination classes of loci each assume a SIM, there appears to be a substantially more recent isolation time for low-recombination loci than for high-recombination loci. Thus, a single SIM (*i.e.*, a historical model with a single isolation time) may not be compatible with both data sets. This led us to develop the second model, a 4-parameter model, which estimates a single isolation time along with the required change in *N*_e_ and rate correction factors for both recombination classes. Confidence intervals for the parameters were estimated by bootstrapping. We find that the 4-parameter model can accommodate the difference in divergence between the two recombination classes by assuming an ancestral *N*_e_ that is only slightly larger than the *N*_e_ of extant *D. simulans*. The results of the analyses indicate that both island species arose at approximately the same time.

## Materials and Methods

### Strains

Genomic DNA was extracted from 5 long-standing isofemale lines of *D. mauritiana*, 4 North American lines of *D. simulans* (courtesy of J. Coyne) and 1 line of *D. sechellia* using a DNeasy kit (Qiagen, Inc.).

### Loci

In addition to 13 loci previously studied [2], [10], 17 loci were sequenced in all of the strains; *D. mauritiana* sequences for 14 of the new loci were used in a separate study on codon usage bias [18]. Most of the new loci were sequenced completely from stop through start codons (see Table 1). *D. melanogaster* sequences for the 17 newly sequenced loci were obtained from Genbank [19]; sources for the 13 previously sequenced loci are given in the related publications. Initial PCR products were purified using a Qiaquick kit (Qiagen, Inc.), and purified PCR products served as templates for cycle-sequencing reactions using the DTCS kit (Beckman-Coulter, Inc.). Sequencing reactions were analyzed using a Beckman-Coulter CEQ 8000 Genetic Analysis System. Internal sequencing primers were designed as needed for complete sequencing of both strands. Sequence data were checked and manually aligned using Sequencher software (Gene Codes, Inc.). Estimates of polymorphism and divergence were based only on sites for which bases were reported for all strains (*i.e.*, any sites with indel variation or unresolved bases were excluded); results were not qualitatively affected by removing such sites. A total of 33,026 aligned sites were analyzed. All sequences have been submitted to Genbank (see Table 1 for accession numbers).

**Table 1:** Loci used in the study.

Locus	Class^a^	L (aligned)^b^	L (analyzed)^c^	Accession Numbers^d^
*128up*	0	1357	1352	EU670439-42, EU670507
*Adh*	0	709	684	
*bap*	0	1270	1270	EU670443-6, EU670508
*Cdc37*	0	1502	1464	EU670447-50, EU670509
*Dnz1*	0	1107	1085	EU670459-62, EU670513
*egh*	0	990	950	EU670463-6, EU670512
*Est6*	0	1529	1521	
*ftz*	0	1389	1376	EU670467-70, EU670514
*Gr47A*	0	1281	1234	EU670471-4, EU670515
*hb*	0	291	235	
*janus*	0	1072	1005	
*kraken*	0	1469	1398	EU670475-8, EU670516
*Or33b*	0	1327	1305	EU670479-82, EU670517
*Pabp2*	0	1192	1112	EU670483-6, EU670518
*Pc*	0	1490	1476	EU670487-90, EU670519
*per*	0	1878	1855	
*Prosα6*	0	1206	1016	EU670491-4, EU670520
*Sxl*	0	297	217	
*Tsp33B*	0	1242	1193	EU670503-6, EU670522
*w*	0	226	154	
*wds*	0	1376	1340	EU670499-502, EU670523-4
*Yp2*	0	1114	1108	
*z*	0	999	930	
*Zw*	0	1323	1272	
*CG15216*	1	1338	1326	EU670451-4, EU670510, EU670525-9
*CG32006*	1	1012	998	EU670455-8, EU670511, EU670530-4
*ase*	1	1067	1058	
*ci*	1	1075	1074	
*ey*	1	1161	1100	
*Sox102F*	1	950	918	EU670495-8, EU670521, EU670535-9

a 0 = HR (high-recombination, 1 = LR (low recombination); *CG15216* is located at polytene map position 40F1; *CG32006*, *ci*, *ey* and *Sox102F* are on the fourth chromosome; *ase* is located at 1B4.

b number of aligned sites.

c number of aligned sites with bases in every sequence.

d accession numbers are for previously unpublished sequences.

### 2-parameter model

For a given trio of species (the island species, *D. simulans* and *D. melanogaster*), three pairwise measures of divergence were obtained. *d̂* *_is_* is the estimate of the average number of pairwise differences between the island species and *D. simulans*; *d̂* *_im_* is this estimate for the island species and *D. melanogaster*; and *d̂* *_sm_* is this estimate for *D. simulans* and *D. melanogaster*. The model assumes an unchanged substitution rate throughout the gene tree, with the exception that a rate change is permitted along the lineage leading to the island species subsequent to isolation. The cause of the rate change (*e.g.*, changing generation time, change in the effectiveness of negative selection), while interesting in its own right, is not important for the model. If *T* is the divergence time for *D. simulans* and *D. melanogaster* lineages, *d*_sm_ = 2*T*μ; this allows an estimate of μ = *d*_sm_/2*T*. Here, *T* is assumed to be 3 mya [1]; estimates of isolation time will scale approximately linearly with *T*. If *t* is the isolation time for *D. simulans* and the island species, *d*_is_ = (1+*f*)*t*μ+*g*θ_s_, where *f* is a substitution rate scalar for the island species, *g* is the ratio of the ancestral population's *N*_e_ to that of extant *D. simulans*, and θ_s_ is the level of polymorphism in *D. simulans*. The term (1+*f*)*t*μ represents the divergence expected since isolation (*i.e.*, *t*μ on the lineages leading to *D. simulans* and *ft*μ on lineages leading to the island species). The term *g*θ_s_ represents the divergence prior to isolation. For the purposes of our analyses, *g* is assumed to be 1.0 for the 2-parameter model, although other values can be used when appropriate. [Kliman et al. [2] used a similar approach, assuming *g* to be 1 and assigning values of *f* based on available data.] Finally, *d*_im_ = (2T−*t*+*tf*)μ. Substituting *d*_sm_/2*T* for μ, the latter formula can be rearranged to give an estimate of *f* in terms of observed divergence estimates and *t*:
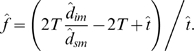
(1)Rearranging the formula *d*_is_ = (1+*f*)*t*μ+*g*θ_s_ to solve for *t*, and substituting the formula for *f* (Eq. 1) and *d*_sm_/2*T* for μ, gives an estimate of *t* based on observed divergence and polymorphism (nucleotide diversity) estimates:
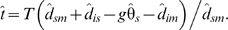
(2)Thus, *t* and *f* are easily estimated simultaneously from polymorphism and divergence data given arbitrary *g* and *T*.

### 4-parameter model

Equations 1 and 2 can be applied to distinct data sets, here genes in low-recombination (LR) and high-recombination (HR) regions, respectively. LR regions, in which recombination is dramatically reduced over an extended region, include the entire fourth chromosome, the tip of the X chromosome and regions near centromeres [20]. Let *t*_0_ and *f*_0_ refer to estimates from HR loci; let *t*_1_ and *f*_1_ refer to estimates from LR loci. Under the SIM, *t*_0_ = *t*_1_. Applying the same subscripts to divergence and polymorphism data for HR and LR loci, and following equation 2,
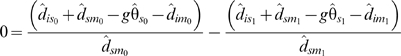
(3)which can be rearranged to yield
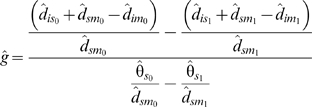
(4)From here, *t̂* is calculated by substituting *ĝ* into equation 2, and *f̂*_0_ and *f̂*_1_ are calculated by substituting *t̂* into equation 1 for HR and LR loci, respectively.

### Confidence intervals for parameter estimates

To obtain 95% confidence intervals for the parameters, sites were sampled with replacement from the full data set to produce 10,000 bootstrap replicates from which parameters were calculated. Bootstrapping of sites was performed three ways: by sampling from all sites regardless of locus or class (HR vs. LR), by sampling while holding the number of sites per class constant, and by sampling while holding the number of sites per locus constant. The three methods were first applied to the original data set. To better capture stochastic variance in time depths of genealogies, which applies here to both polymorphism and divergence, all three methods were applied to data sets for which loci were sampled with replacement within recombination class. One hundred bootstrapped locus lists were generated, and each was used for 100 bootstraps of sites. As expected, confidence intervals were wider when loci, as well as sites, were re-sampled. We found that 100 re-samplings of loci is sufficient to construct the distributions of parameter estimates, as the distributions are essentially the same as those derived from 1,000 re-samplings.

## Results and Discussion

### DNA sequence variation

Contributions of individual loci to measures of divergence and polymorphism for all sites are shown in Table 2. Two estimates of θ_s_ are shown: an estimate based on average pairwise nucleotide diversity, θ̂_π_ [21], and Watterson's estimator, θ̂*_W_*, which is based on the number of polymorphic sites [22]. As expected, polymorphism at all LR loci is low for both *D. simulans* and *D. mauritiana* (Tables 2 and 3); this also suggests that these regions of low recombination are shared by the species. It is worth noting that divergence at the LR locus *CG15216*, which is located near the centromere of chromosome 2, is not reduced within the *D. simulans* complex. Analyses were performed both with and without this locus. It is immediately apparent that divergence between *D. simulans* and the island species is reduced at LR loci (Students *t* test: *p* = 0.00396 for *D. mauritiana*, *p* = 0.00243 for *D. sechellia*; when *CG15216* is excluded, *p* = 0.00396 for *D. mauritiana*, *p* = 0.00156 for *D. sechellia*; corresponding Mann-Whitney U tests were also significant at *p*<0.01). However, divergence is similar for the two recombination classes between either species and *D. melanogaster* (Table 3); differences are not statistically significant and the means for LR loci exceed those for HR loci.

**Table 2:** Divergence and *Drosophila simulans* polymorphism.

Locus	Class^a^	θ̂_π_	θ̂*_W_*	*d̂* *_sm_*	*D. mauritiana*		*D. sechellia*	
					*d̂* *_is_*	*d̂* *_im_*	*d̂* *_is_*	*d̂* *_im_*
*128up*	0	10.0000	8.1818	45.5000	24.5000	51.4000	29.0000	51.0000
*Adh*	0	4.6000	5.2555	18.3333	9.0556	20.6667	11.5000	19.5000
*bap*	0	14.8333	14.7273	43.5000	13.4000	40.2000	22.5000	48.0000
*Cdc37*	0	15.6667	15.2727	56.7500	18.6500	59.2000	20.2500	55.0000
*Dnz1*	0	11.5000	12.5455	48.0000	22.0000	49.6000	27.7500	47.0000
*egh*	0	6.6667	6.5455	47.2500	9.6000	47.6000	9.5000	49.0000
*Est6*	0	38.3333	37.6364	77.0577	36.8750	76.4487	48.0000	77.1538
*ftz*	0	9.5000	9.2727	49.2500	16.8500	51.2000	28.0000	51.0000
*Gr47A*	0	0.5000	0.5455	74.0000	26.6500	80.4000	32.5000	86.0000
*hb*	0	1.3333	2.2076	5.6667	1.0000	5.3333	0.6667	5.0000
*janus*	0	19.3333	19.3333	52.1111	17.8889	49.3704	30.3333	57.4444
*kraken*	0	7.5000	8.1818	50.5000	17.2000	49.0000	24.2500	54.0000
*Or33b*	0	17.0000	18.5455	70.2500	29.2500	64.4000	40.2500	72.0000
*Pabp2*	0	5.5000	5.4545	49.2500	8.8000	50.6000	12.5000	54.0000
*Pc*	0	4.1667	3.8182	44.2500	7.9500	47.0000	9.7500	51.0000
*per*	0	20.2667	22.3358	64.3333	33.0278	74.6667	30.7778	71.6667
*Pros*α*6*	0	9.1667	8.7273	50.7500	17.7500	52.6000	18.2500	57.0000
*Sxl*	0	1.9848	1.9868	7.1667	1.6786	7.4286	4.4167	11.0000
*Tsp33B*	0	4.3333	3.8182	47.5000	8.9000	51.6000	12.5000	53.0000
*w*	0	4.4444	4.4152	8.5556	3.3889	6.8333	3.7778	8.3333
*wds*	0	0.0000	0.0000	53.0000	23.6000	51.0000	22.0000	46.0000
*Yp2*	0	1.2000	1.3139	28.6667	4.1667	30.8333	4.1667	30.1667
*z*	0	7.4667	7.4453	35.3333	8.8333	34.0000	12.0000	35.6667
*Zw*	0	4.3788	3.3114	43.7167	10.2500	43.8000	16.2500	49.8000
*CG15216*	1	0.0000	0.0000	62.0000	12.0000	60.0000	18.0000	68.0000
*CG32006*	1	0.5000	0.5455	60.2500	5.0500	64.8000	6.2500	64.0000
*ase*	1	0.0000	0.0000	26.8333	2.6000	28.4333	2.0000	25.8333
*ci*	1	0.2222	0.3679	54.1111	6.2778	54.1667	5.1111	53.0000
*ey*	1	1.0476	1.2245	41.0000	4.1000	43.1000	3.2500	42.0000
*Sox102F*	1	0.5000	0.5455	60.2500	5.4500	61.2000	6.2500	63.0000

a 0 = HR locus, 1 = LR locus.

**Table 3:** Polymorphism and Divergence (per site) in low-recombination and high-recombination loci (unweighted average).

Class	L^a^	*D. simulans*		*D. mauritiana*		*d̂* *_sm_*	*D. mauritiana*		*D. sechellia*	
		θ̂_π_	θ̂*_W_*	θ̂_π_	θ̂*_W_*		*d̂* *_is_*	*d̂* *_im_*	*d̂* *_is_*	*d̂* *_im_*
HR	26552	0.00882	0.00899	0.00683	0.00718	0.04021	0.01353	0.04054	0.01743	0.04301
LR	6474	0.00037	0.00043	0.00060	0.00067	0.04763	0.00535	0.04889	0.00604	0.04933
LR without CG15216	5148	0.00044	0.00052	0.00072	0.00080	0.04780	0.00461	0.04962	0.00453	0.04894

a number of sites analyzed (see Table 1).

These findings indicate that the reduction in divergence within the complex at LR loci is probably not due to greater selective constraint. The pattern also contrasts with findings in primates [23], where a positive correlation between divergence and recombination rate is observed in several species pairs, including distantly related pairs for which the contribution of the coalescent in the common ancestral pair should be minor (*i.e.*, in accordance with the model used by Birky and Walsh [24]). Thus, while recombination rate and mutation rate may be associated in primates, our data are not explained by such an association. Ometto et al. [25] also observed a positive correlation between recombination rate and intron divergence of *D. melanogaster* and *D. simulans* (although not for divergence in intergenic regions), which may have a non-neutral explanation; however, as noted above, we do not observe a corresponding pattern in our data when comparing the two recombination classes.

### 2-parameter model

**Figure 1:**
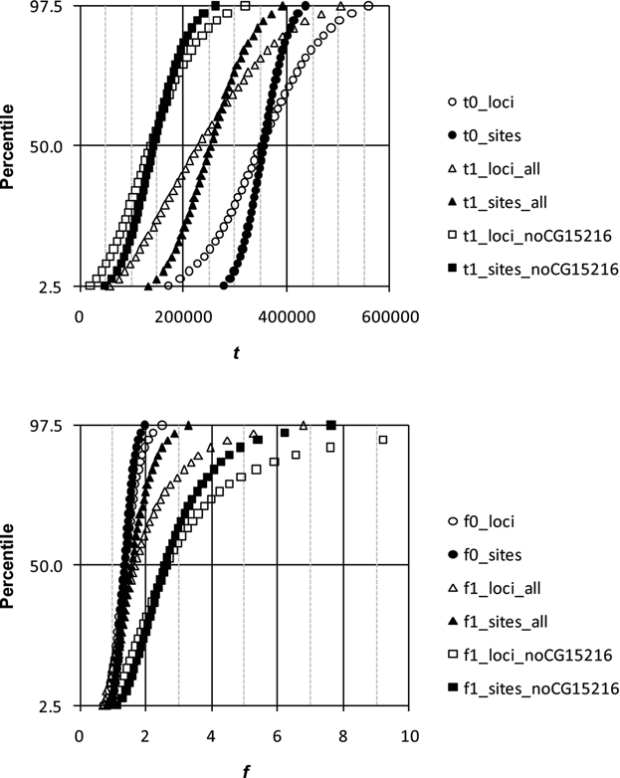
Distribution of parameters estimated from 10,000 bootstrapped data sets using the 2-parameter model for *D. simulans* and *D. mauritiana*. Shown are distributions using θ̂_π_ as the estimate of *D. simulans* polymorphism. Suffixes in legend: loci, bootstrapping among both loci and of sites within loci; sites, bootstrapping of sites, but not loci; all, all LR loci; noCG15216, *CG15216* excluded from LR loci.

**Figure 2:**
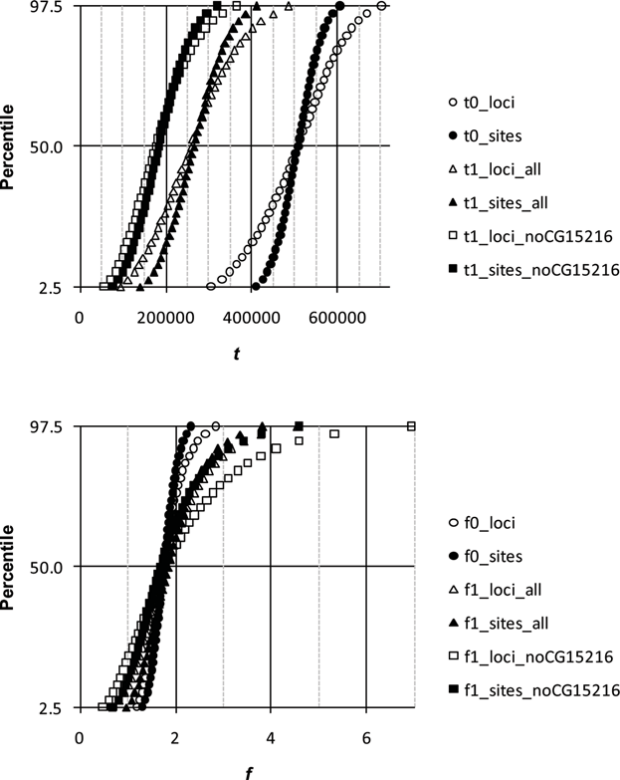
Distribution of parameters estimated from 10,000 bootstrapped data sets using the 2-parameter model for *D. simulans* and *D. sechellia*. See Figure 1 for details.

Estimates of *t* and *f* for HR and LR loci are shown in Table 4. Distributions of the parameter estimates, as obtained by bootstrapping, are shown for estimates using *D. simulans* θ̂_π_ in Figures 1 and 2 for the pairs *D. simulans*/*D. mauritiana* and *D. simulans*/*D. sechellia*, respectively. Distributions for estimates using θ̂*_W_* were nearly identical (data not shown). For both species pairs, isolation times are more recent for LR loci (∼250–270 kya for both species) than for HR loci (∼350 kya for *D. simulans*/*D. mauritiana* and ∼500 kya for *D. simulans*/*D. sechellia*), although the confidence intervals are broad. The method used to bootstrap sites had virtually no effect on the shapes of the distributions; therefore, only distributions for bootstrapping while holding sites per locus constant are shown. However, when loci were sampled with replacement (open markers in Figures 1 and 2), distributions were broader than when loci were not re-sampled (closed markers). This indicates that to better capture stochastic error with these data, loci must be re-sampled.

**Table 4:** Estimates of *t* and *f* using the 2-parameter model.

	*D. mauritiana*			*D. sechellia*		
	HR	LR	LR without CG15216	HR	LR	LR without CG15216
*t̂* (using θ̂_π_)	356125	255735	147898	509737	268053	188114
*f̂* (using θ̂_π_)	1.3854	1.5591	2.5487	1.7590	1.8374	1.7090
*t̂* (using θ̂*_W_*)	352755	251661	142781	506367	263978	182997
*f̂* (using θ̂*_W_*)	1.3891	1.5682	2.6042	1.7641	1.8503	1.7288

As noted earlier, levels of DNA sequence divergence among closely related species vary widely across loci. This is probably due, in part, to differences in selective constraint. It may also reflect stochastic variance in the time depths of the coalescent of the ancestral population into which the lineages from the extant species descend. Thus, the low divergence within the *D. simulans* complex at fourth chromosome loci could simply reflect stochastic variance in the ancestral population; it could be coincidental that *ase*, located at the tip of the X chromosome, also shows low divergence within the complex. However, low divergence at LR loci could also reflect a shallow coalescent in the ancestral population due to Hill-Robertson effects predicted for regions of low recombination (*e.g.*, hitchhiking following selective sweeps, background selection) [12], [13], [26], [27]. Still, if reduced divergence at LR loci reflects selective sweeps, and if gene flow ceased at the same time for all loci, then we should obtain an estimate of the isolation time (*t*) that is similar to that for HR loci. Regardless of the bootstrapping approach, while there is overlap in distributions of bootstrapped estimates of *t* for LR and HR loci for both species pairs, the distributions are clearly significantly different (Mann-Whitney U test, *p*<10^−6^).

One interpretation of these findings is that LR loci were, in fact, genetically isolated more recently – *i.e.*, gene flow between the incipient island species and incipient *D. simulans* continued longer for LR loci than it did for HR loci. If hybridization were occurring subsequent to initial geographic isolation of the species, this explanation would require that HR loci face a greater barrier to gene flow than do LR loci. This is possible if loci contributing to hybrid incompatibility are numerous and broadly distributed across the genome, but absent from the regions of low recombination sampled in this study. Coyne and Berry [11] have shown that the D. simulans fourth chromosome does not harbor loci contributing to sterility in hybrids with *D. mauritiana* and *D. sechellia*. In their study of *D. mauritiana* introgression into a *D. simulans* background, True, Laurie and Weir [4] found that markers associated with hybrid sterility were distributed broadly throughout the genome; however, introgression of markers located at the tip of the X chromosome did not reduce fertility. Similarly, introgression of the tip of the *D. mauritiana* X chromosome into a *D. sechellia* background also did not reduce fertility [8] (D.C. Presgraves, personal communication).

Under this interpretation of the findings, low divergence at LR loci could reflect gene flow, *i.e.*, rejection of the SIM, whereby gene flow ceased at the same time for all loci, perhaps at the time of initial geographic isolation. We applied a pair of tests of the SIM that allow stochastic variance to the species pair for which polymorphism was estimated for all thirty loci (*D. simulans* and *D. mauritiana*) and were unable to reject a SIM (*p* = 0.6150 [28]; *p* = 0.1020 [2]). However, it should be noted that these tests of the SIM are conservative.

### Sensitivity of 2-parameter model isolation time estimates to *f*

**Figure 3:**
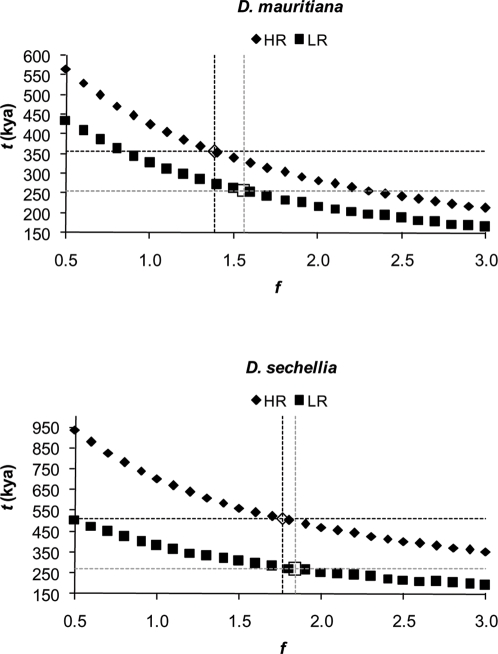
Sensitivity of isolation time estimates to substitution rate along the island species lineage. Open markers (intersected by dashed horizontal and vertical lines) indicate estimates of *t* corresponding to *f* under the 2-parameter model. All other points indicate values of *t* corresponding to arbitrary values of *f* ranging from 0.5 to 3.0. HR, high-recombination loci; LR, low-recombination loci.

Given the expectation that *d*_is_ = (1+*f*)*t*μ+*g*θ_s_, *t* can be estimated from polymorphism and divergence:
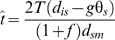
(5)Since not all data sets will include loci in two distinct classes that allow appropriate use of the 4-parameter model, and since the confidence intervals for *f* in our analyses are broad, it is worth exploring the effect of varying *f* on estimates of *t*. Figure 3 shows estimates of *t* under the 2-parameter model for LR and HR loci. Clearly, estimates of *t* are more sensitive to change at low values of *f*. Although the limit of *t* as *f* approaches infinity is zero, the curve is flatter. Since both island species have diverged more from *D. melanogaster* than has *D. simulans*, it is unlikely that true values of *f* are below 1.0 for either.

### 4-parameter model

An alternative interpretation of the findings from analyses using the 2-parameter model allows for gene flow to cease for all loci at the same time – *i.e.*, it allows for a single SIM applicable to all loci. It requires that the ratio of coalescent time of (i) lineages descending from *D. simulans* and the island species within the ancestral population to (ii) lineages within extant *D. simulans* be greater for HR loci than for LR loci. This is expected if coalescent time is markedly reduced in LR regions (and essentially the same in extant *D. simulans* and the common ancestral population) and if the *N*_e_ of the ancestral population is greater than that of extant *D. simulans* (*i.e.*, if this ratio, *g*, is greater than 1). As *N*_e_ of the ancestral population increases, the contribution of the coalescent in the common ancestor to total divergence increases proportionately.

Birky and Walsh [24] predicted that recombination rate should have little effect on divergence; however, they noted the important exception: that the coalescent in the common ancestor becomes relevant when divergent taxa are very closely related. Consistent with Birky and Walsh [24], hitchhiking and pseudohitchhiking, by dramatically reducing *N*_e_ in the common ancestral and extant populations, will reduce both total divergence between closely related species and nucleotide diversity within species in regions of low recombination.

**Figure 4:**
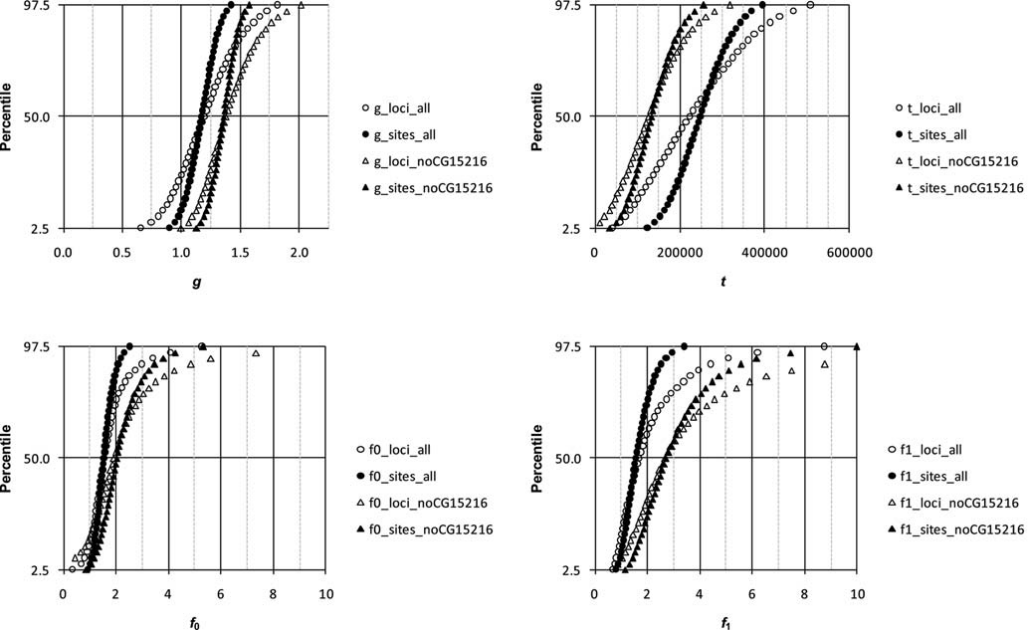
Distribution of parameters estimated from 10,000 bootstrapped data sets using the 4-parameter model for *D. simulans* and *D. mauritiana*. See Figure 1 for details.

**Figure 5:**
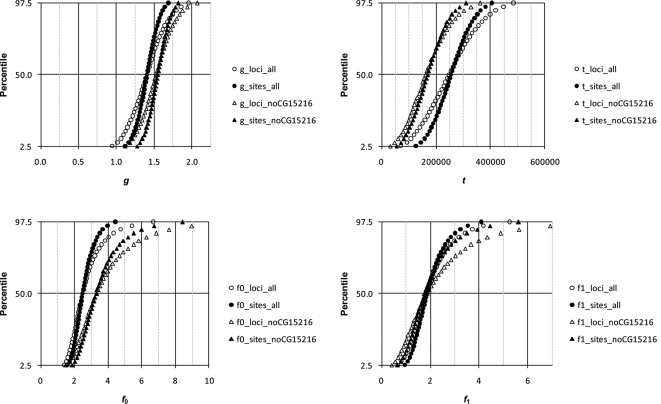
Distribution of parameters estimated from 10,000 bootstrapped data sets using the 4-parameter model for *D. simulans* and *D. sechellia*. See Figure 1 for details.

Estimates of *t*, *g*, *f*_0_ and *f*_1_ are shown in Table 5. Distributions of the parameters (using θ̂_π_) are shown in Figures 4 and 5 for analyses of *D. simulans*/*D. mauritiana* and *D. simulans*/*D. sechellia*, respectively. The isolation times of both species are estimated at ∼250 kya. The large differences obtained for estimates of *t* for LR and HR loci under the 2-parameter model are reconciled by a very modest decrease in the *N*_e_ of extant *D. simulans* since isolation, with *g* estimated at ∼1.2 for *D. mauritiana* and ∼1.4 for *D. sechellia* using all loci. If the two island species arose at the same time, then *g* should be similar for both species, and there is considerable overlap in the confidence intervals. However, it is worth noting that if the species arose at different times, *g* can be different since *N*_e_ can change over time for the ancestral population.

**Table 5:** Estimates of *t*, *g*, *f*_0_ and *f*_1_ using the 4-parameter model.

	*D. mauritiana*		*D. sechellia*	
	All loci	CG15216 removed	All loci	CG15216 removed
*t̂* (using θ̂_π_)	251950	137942	258939	172736
*ĝ* (using θ̂_π_)	1.1693	1.3545	1.4075	1.5475
*f̂*_0_ (using θ̂_π_)	1.5447	1.9949	2.4942	3.2398
*f̂*_1_ (using θ̂_π_)	1.5676	2.6605	1.8668	1.7721
*t̂* (using θ̂*_W_*)	247149	130878	253160	164665
*ĝ* (using θ̂*_W_*)	1.1706	1.3585	1.4091	1.5521
*f̂*_0_ (using θ̂*_W_*)	1.5553	2.0486	2.5283	3.3496
*f̂*_1_ (using θ̂*_W_*)	1.5786	2.7502	1.8866	1.8099

Thus, it is possible to reconcile the differences in divergence for LR and HR loci by allowing for an ancestral *N*_e_ that exceeds that of extant *D. simulans*. In a constant *N* model, two randomly chosen lineages in a population should coalesce in *pN* generations (where *p* is the effective ploidy). Therefore, polymorphism (θ_s_) in extant *D. simulans* should be 2*pN*_e_μ. Working back from the time of initial isolation, lineages in the ancestral population should have a mean coalescent time of *gpN*_e_ generations. If, as introduced above, θ is reduced in LR loci due to strong regional *N*_e_-reducing effects of selection, both in extant *D. simulans* and in the ancestral population, then if *g* exceeds 1, divergence would be disproportionately greater in HR genes than would be expected when *g* is 1. In our analyses, *g* does not have to be much greater than 1.0 to explain the difference in divergence.

On the other hand, this reconciliation appears less reasonable when *CG15216*, the near-centromeric locus, is left out of the analyses. In contrast to the other five LR loci, divergence (per bp) between *D. simulans* and the island species at *CG15216* is only slightly reduced (*D. mauritiana* HR loci, average *d* = 0.01353±0.00127 [s.e.m.]; LR locus *CG15216*, *d* = 0.00905; other five LR loci, average *d* = 0.00461±0.00067; *D. sechellia* HR loci, average *d* = 0.01743±0.00164; LR locus *CG15216*, *d* = 0.01358; other five LR loci, average *d* = 0.00453±0.00094). When *CG15216* is excluded, isolation times are estimated to be more recent for LR loci under the 2-parameter model (Table 4) and for all loci under the 4-parameter model (Table 5), *i.e.*, between 100 and 200 kya. However, estimates of substitution rate correction factors rise quite a bit under the 4-parameter model. Therefore, reconciliation of the different levels of divergence at LR and HR with the SIM intrinsic to the 4-parameter model requires much more highly elevated substitution rates along the lineages leading to the island species than are required by allowing different isolation times for the two classes of loci.

### Inclusion of additional African *D. simulans* data

As noted earlier, the seventeen newly sequenced loci were sampled in four North American lines of *D. simulans*. African lines were generally included in the data sets of the thirteen previously analyzed loci. Given the possibility that inclusion of African lines could lead to increased estimates of θ, we repeated some of the analyses with *D. simulans* sequence data included in the Drosophila Population Genomics Project [29]. The “vertical multiple alignment” (VMA) files of syntenic assemblies for two Madagascar lines (md106 and md199) and one line from Kenya (c167.4) were downloaded from http://www.dpgp.org. FASTA files for each chromosome arm were generated using the perl program (vma2fasta2.pl) provided at the site. This program allows filtering of the VMA files in order to include only base calls that exceed a threshold quality score; for our analyses, this was set to 30. No data were available for the near-centromere locus *CG15216* or for the chromosome 4 loci; however, little polymorphism was observed at these loci in the North American lines (or in *D. mauritiana*), so the lack of data from African lines probably does not affect our analyses.

For *egh*, base calls with quality scores exceeding 30 were limited to 23.8% of sites in c167.4, 12.6% in md106 and 1.0% in md199. For *ftz* and *pc*, only 13.0% and 11.8% of sites were called, respectively, in c167.4. No bases were called for *Prosα6* in any of the African lines. Since we only include sites that are resolved in all strains, we excluded these sequences. The analyses were first repeated with data from c167.4 (when available) and the Madagascar strain with the most base calls. They were then repeated with the single African strain with the most base calls. The latter always included more sites, but decreased the number of pairwise contrasts with African lines. As before, analyses were performed both with and without *CG15216*. Bootstrapping was performed by resampling loci and sites within loci as described earlier.

Parameter estimates, with percentage change from analyses without the added data, are shown in Table 6. In general, inclusion of African lines increased estimates of θ, but did not increase estimates of divergence from *D. melanogaster*, indicating that the increased estimates of θ do not reflect any systematic sequencing error. Instead, these estimates might reflect a decrease in θ in North American populations, consistent with a proposed population bottleneck [30]. Consequently, estimates of *g* under the 4-parameter model, while still above 1.0, are reduced relative to those estimated prior to inclusion of the data from African lines. However, the isolation time estimates under the 4-parameter model are nearly unchanged (with percentage changes ranging from +1.2% to +4.6%). The isolation time estimates for HR loci under the 2-parameter model are somewhat reduced. However, these remain significantly higher (Mann-Whitney U test, p<10^−6^) than estimates for LR loci. Therefore, our findings are qualitatively unchanged. Perhaps of greater interest, it appears that a single isolation time estimate for HR and LR loci can be reconciled under a SIM by allowing an even more modest change in *N*_e_ than indicated by analyses without the added data from African lines.

**Table 6:** Parameter estimates following inclusion of additional *D. simulans* data from African lines.

Analysis	*t*_0_ (2-p)^a^	*f*_0_ (2-p)^a^	*t* (4-p)	*g* (4-p)	*f*_0_ (4-p)	*f*_1_ (4-p)
*D. mauritiana*, 1–2 Afr^b^	277538 (−22.1%)	1.2971 (−6.4%)	255046 (+1.2%)	1.0299 (−11.9%)	1.3233 (−14.3%)	1.5610 (−0.4%)
*D. mauritiana*, 1 Afr^c^	297026 (−16.6%)	1.2893 (−6.9%)	254423 (+1.0%)	1.0578 (−9.5%)	1.3378 (−13.4%)	1.5624 (−0.3%)
*D. mauritiana*, 1–2 Afr, no *CG15216*	same	same	142834 (+3.5%)	1.1794 (−12.9%)	1.5774 (−20.9%)	2.6044 (−2.1%)
*D. mauritiana*, 1 Afr, no *CG15216*	same	same	141964 (+2.9%)	1.2103 (−10.6%)	1.6053 (−19.5%)	2.6143 (−1.7%)
*D. sechellia*, 1–2 Afr	378903 (−25.7%)	2.1981 (+24.9%)	264630 (+2.2%)	1.1521 (−18.1%)	2.7154 (+8.9%)	1.8483 (−1.0%)
*D. sechellia*, 1 Afr	421821 (−16.7%)	1.9045 (+11.4%)	263221 (+1.7%)	1.2151 (−13.7%)	2.4495 (−1.8%)	1.8528 (−0.7%)
*D. sechellia*, 1–2 Afr, no *CG15216*	same	same	180674 (+4.6%)	1.2639 (−18.3%)	3.5125 (+8.4%)	1.7383 (−1.9%)
*D. sechellia*, 1 Afr, no *CG15216*	same	same	178829 (+3.5%)	1.3296 (−14.1%)	3.1335 (−3.3%)	1.7459 (−1.5%)

a parameter estimates calculated using θ̂_π_; estimates under the 2-parameter model for HR loci without *CG15216* are the same as those with this locus.

b parameter estimates using c167.4 (when available) and either md106 or md199.

c parameter estimates using either c167.4, md106 or md199 (see text).

### Sensitivity of parameter estimates to site type

The thirty loci used in this study differ in the proportion of synonymous, replacement and noncoding sites. It is reasonable to ask whether parameter estimates change if analyses are limited to a specific type of site. Parameter estimates for specific site types (using θ̂_π_) are given in Table 7. Estimates of *t* from replacement sites tend to be lower, with concomitantly higher estimates of *f*. For the 4-parameter model, estimates of *f* are sometimes exceedingly high, particularly for replacement sites. If this simply reflected a marked reduction of the efficacy of purifying selection on amino acid sequences in the island species (perhaps due to reduced *N*_e_), the model might be acceptable. However, given such high estimates of *f* from the current data for replacement sites, it is probably inadvisable to accept the corresponding estimates of *t*.

**Table 7:** Parameter estimates using synonymous, replacement or noncoding sites^a^.

Sites	*t*_0_ (2-p)^b^	*f*_0_ (2-p)	*t*_1_ (2-p)	*f*_1_ (2-p)	*t* (4-p)^c^	*g* (4-p)	*f*_0_ (4-p)	*f*_1_ (4-p)
*D. mauritiana*								
Synonymous	342909	1.3928	397590	1.6909	397590	0.9178	1.3387	1.6909
Replacement	273108	2.0687	131234	1.6800	116719	1.3626	3.5006	1.7646
Noncoding	398895	1.1844	252692	1.3836	246834	1.2418	1.2980	1.3927
*D.m.* excluding *CG15216*								
Synonymous	342909	1.3928	241935	2.5200	241935	1.1518	1.5567	2.5200
Replacement	273108	2.0687	77479	3.3600	53402	1.5094	6.4656	4.4240
Noncoding	398895	1.1844	144175	2.2525	131156	1.4257	1.5609	2.3769
*D. sechellia*								
Synonymous	514079	1.5747	433735	1.0000	433735	1.1208	1.6811	1.0000
Replacement	557945	2.1141	164042	2.7560	123744	2.0068	6.0234	3.3278
Noncoding	478094	1.8391	235385	2.3725	225659	1.4014	2.7777	2.4317
*D.s.* excluding *CG15216*								
Synonymous	514079	1.5747	387097	0.5000	387097	1.1909	1.7632	0.5000
Replacement	557945	2.1141	116219	2.5933	61854	2.1503	11.0497	3.9938
Noncoding	478094	1.8391	122330	3.3810	104146	1.5946	4.8518	3.7967

a parameter estimates calculated using θ̂_π_.

b 2-parameter model.

c 4-parameter model.

### Relevance to efforts to infer the history of the *D. simulans* species complex

The evolutionary history of the *D. simulans* complex has been debated (*e.g.*, [2], [31]), meriting attention at least in part because of the importance of the complex as a model for speciation. A generally accepted strict bifurcating phylogeny has not emerged. In general, *D. sechellia* is more diverged at the DNA sequence level from the other two species, although this must be influenced to some extent by the higher substitution rate in *D. sechellia*. Our analyses indicate that the two island species arose at about the same time. In comparison to previous estimates scaled by a 3 mya divergence time for *D. melanogaster* and the *D. simulans* complex, the estimated isolation time for *D. mauritiana* is essentially unchanged (∼250 kya). However, this date is considerably more recent than that estimated previously for *D. sechellia* [2]. This may be contributing to the difficulty in reconstructing the phylogeny of this species complex. Given that the dates are similar for the two island species, it also lends support to the possibility that they arose from a common ancestor itself isolated from the mainland species, a scenario previously suggested by Caccone et al. [32].
